# Predicting the number of grazing days from milk Fourier-transform mid-infrared spectral analysis

**DOI:** 10.3168/jdsc.2025-0954

**Published:** 2026-04-20

**Authors:** Killian Dichou, Didier Veselko, Antonino Marvuglia, Hélène Soyeurt

**Affiliations:** 1 TERRA Research and Teaching Centre, Gembloux Agro-Bio Tech, University of Liège, 5030 Gembloux, Belgium; 2 Comité du Lait, B-4651 Battice, Belgium; 3 Luxembourg Institute of Science and Technology (LIST), 4362 Esch-sur-Alzette, Luxembourg

## Abstract

Grazing provides multiple benefits for dairy farming, including reduced feeding costs, improved animal welfare, environmental services, and enhanced milk composition. Current certification schemes often require a minimum number of grazing days, typically verified through grazing calendars, which are time consuming for farmers and costly for industry controls. This study evaluates Ind_Herbage, an indicator derived from bulk milk Fourier-transform mid-infrared spectra to predict the probability of herbage consumption, as a proxy for grazing activity in Wallonia, southern Belgium. The algorithm detects grazing periods by analyzing temporal changes in Ind_Herbage values using a smoothed derivative-based approach with a winter correction to avoid false positives from feed supplementation. Applied to 2 yr of data from 72 Walloon dairy farms (approximately 10,000 milk records per year), the method identified an average of 212 grazing days in 2023 and 221 in 2024, with 97% of farms exceeding the 120-d requirement. The number of grazing periods detected per farm (4–5 on average) was well aligned with regional climatic conditions. These results demonstrate the potential of Ind_Herbage to provide an automatic, large-scale assessment of grazing days, offering a practical alternative to traditional grazing calendars for both farmers and dairy industries.

## Highlights

• Practical use of a grazing indicator based on milk FT-MIR spectral analysis.
• Possibility to count the number of grazing days using the derivative of a grazing indicator.
• Detection rate for 120 grazing days requirement at 97.2%.
• Scalable algorithm of detection on tank milk data.

## Graphical Abstract

Grazing offers multiple benefits for dairy farming, including lower feeding costs, improved animal welfare, environmental services, and enhanced milk composition. Current certification schemes usually require a minimum number of grazing days, verified through grazing calendars that are time-consuming for farmers and costly to control. This study evaluates Ind_Herbage, an indicator derived from bulk milk Fourier-transform mid-infrared (FT-MIR) spectra, to estimate the probability of herbage consumption as a proxy for grazing activity in Wallonia, southern Belgium. Grazing periods were identified by analyzing temporal changes in Ind_Herbage using a smoothed derivative-based approach with a winter correction to limit false positives due to feed supplementation. Applied to 2 years of data from 72 dairy farms (about 10,000 milk records annually), the method detected an average of 212 grazing days in 2023 and 221 in 2024, with 97% of farms exceeding the 120-day requirement. These results highlight the potential for Ind_Herbage as a practical, automated alternative to traditional grazing calendars.

**Figure:**
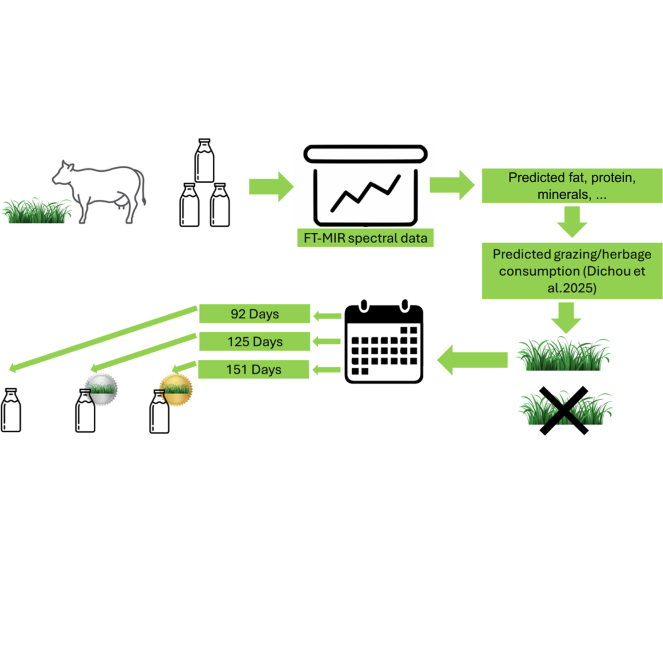

In Europe, grazing is a common farm management practice (Dufrasne Isabelle and Françoise Lessire, 2017). It yields several economic advantages by fostering the use of locally available feed and thereby reducing feeding costs, especially during periods of high stock market price fluctuations (Perry et al., 2014; Hanrahan et al., 2018). Grazing is also frequently associated with improved animal welfare, as it allows cattle to express natural behaviors and improves their health by, for instance, reducing the risk of locomotion-related illnesses (McLellan et al., 2022; Tillack et al., 2024). From an environmental perspective, pastures are well known for their role as ecosystem service providers, particularly in carbon sequestration (Phukubye et al., 2022; Intergovernmental Panel On Climate Change, 2023), as well as their biodiversity preservation functions (Lengyel et al., 2023; Elliott et al., 2024). These are the primary benefits of pastures highlighted by the Common Agricultural Policy (**CAP**) and appearing in the CAP 2024–2027 guidelines (European Commission 2023). Specifically in dairy cattle, grazing brings significant benefits to milk composition, in many cases improving the content of n-3 fatty acids, conjugated linoleic acid, and polyunsaturated fatty acids in milk fat (Techeira et al., 2023; Rooney et al., 2025).

Some labels for milk and dairy products already impose specific grazing requirements. For instance, “Lait Campina,” “Albert Heijn Goudse,” and “Pro Weideland Milch” require 120 grazing days with 6 h of grazing per day; “Le brie belge” requires 180 grazing days from milk collected by “Marguerite Happy Cow,” and “Lait Les 20 fermes” has a minimum requirement of 200 grazing days.

To ensure that these requirements are met, common practice in Europe relies on grazing calendars requested by dairy industries. Grazing management already requires considerable planning, time, and experience to optimize available resources (Deming et al., 2018; Hogan et al., 2023). To facilitate this assessment, indicators based on milk response to grazing have been developed (Birkinshaw et al., 2024; Dichou et al., 2025) and could provide an automatic method to replace grazing calendars. On the industry side, the use of automatic methods based on these indicators could reduce the cost of farm controls to evaluate the reliability of compiled grazing calendars. On the farmers' side, it could replace the grazing calendars themselves, which are time consuming and increase the farmer's overall mental workload. This study aims to evaluate the potential of a pasture detection indicator (termed **Ind_Herbage**) applied to bulk milk data and proposes an algorithm to automatically count the number of days cows spend on pasture.

Ind_Herbage is an indicator based on Model 4 of Dichou et al. (2025), which was identified as the most promising model. It represents the probability of belonging to the herbage consumption group—a group of observations that presented values of “time on pasture,” “herbage in the ration,” and “estimated rumen fill from the ration” associated with grazing. The indicator is based on several milk component predictions (such as the predicted content of C18:1 *trans* and conjugated linoleic acid), acidity traits (such as predicted pH and predicted titratable acidity), protein-related traits (such as predicted whey proteins or predicted protein efficiency), predicted mineral content for Ca, P, K, and Mg, and other parameters. All traits were predicted from milk Fourier-transform mid-infrared (**FT-MIR**) spectra, which were generated by analyzing bulk milk using MilkoScan spectrometers (Foss Electric A/S, Hillerød, Denmark) at the milk laboratory “Comité du Lait” (Battice, Belgium) and standardized using the method developed by Grelet et al. (2017). The main hypothesis of this study is that the proposed indicator can be used as a proxy for grazing, although it more accurately reflects herbage consumption. This study incorporates temporal dynamics into the analysis to estimate grazing periods on a farm-by-farm basis, moving beyond indicator development toward practical application.

Ind_Herbage was computed on available data to estimate the number of grazing days per year. The database is the same validation subset used in Dichou et al. (2025), but this study focuses on a larger temporal scale, as a whole year is used to count the number of days. This dataset, “DT1,” comprises 72 Walloon farms over 2 yr of observations, that is, 144 farm-year observations, corresponding to 9,557 analyses in 2023 and 10,112 analyses in 2024. These analyses are related to milk payments, with bulk milk being collected every 1 to 3 d at each farm. The algorithm was also tested on a smaller dataset, “DT2,” with month-to-month validation data for 8 different farms and information on the percentage of added flaxseed-rich feed. It comprises 66 farm-month observations in 2024. On a larger scale, our approach was also applied to the whole Walloon database “DT3” as a means to assess its potential for large-scale screening. The DT3 therefore includes both DT1 and DT2, containing 2,296 farm-year observations for 2023 and 2,213 observations for 2024 in total. All farms included in DT1 and DT2 incorporate grazing in their management. Data from farms managed without grazing were not available in Wallonia.

To count the number of days that could be flagged as grazing days, the simplest approaches using a fixed threshold or a moving threshold were discarded due to scale differences between farms. The indicator is interpreted as the effect of feed on milk composition and is based on the assumption that milk composition is not only affected by diet (as even the composition of herbage can be quite diverse; Peyraud and Delagarde, 2013; Suter et al., 2021) but also by genetics, farm conditions, and external conditions.

Therefore, our approach is based on detecting substantial increases or decreases in the indicator. To this end, the evolution of this indicator is treated as a signal evolving throughout the year, and the derivative is used to measure the variation of the signal.

As with signals from specific milk components, such as fat content or protein content detected via milk infrared analysis, temporal analysis of signal fluctuation requires smoothing, especially if the goal is to use the signal's derivative. Several methods are used in milk FT-MIR spectrometry to reduce noise around the signal (Grelet et al., 2017). The goal is to reduce noise without losing too much information. Given our data availability, a moving average was performed 7 d before the current observation. Therefore, not all analyses were averaged over the same number of observations, because the number of analyses for a farm within those 7 d can vary between 1 and 7, depending on how frequently milk is collected. In DT3, the complete database, 92.0% of the averages were made on 3 to 4 observations in 2023 and 90.8% in 2024.

By default, the derivative is computed between 2 consecutive analyses if data availability permits. In cases of field-related problems (sample collection issues, calibration problems, machine unavailability, sample loss, and so on), a rule was set to ensure a maximum of 7 d between 2 analyses to compute the derivative. Once all derivatives were computed, we created a flag for pasture. After several trials, a seemingly acceptable range was defined as 4 consecutive positive values of the derivative to indicate the beginning of a grazing interval and 4 consecutive negative values of the derivative (as long as negative values persist) to indicate the end of a grazing period. This brings the range of pasture detection from a minimum of 4 d to a maximum of 28 d to begin or stop grazing. We automatically checked the length of the “starting” and “stopping” period for each grazing period. Looking at the third quartile, 75% of the observations display approximately 2 wk (12 to 15 d) for the 4 starting points (4 consecutive positive derivatives) and for the 4 stopping points (last 4 consecutive negative derivatives after a starting point). An example of period detection for one farm in the dataset is presented in Figure 1.

**Figure 1:**
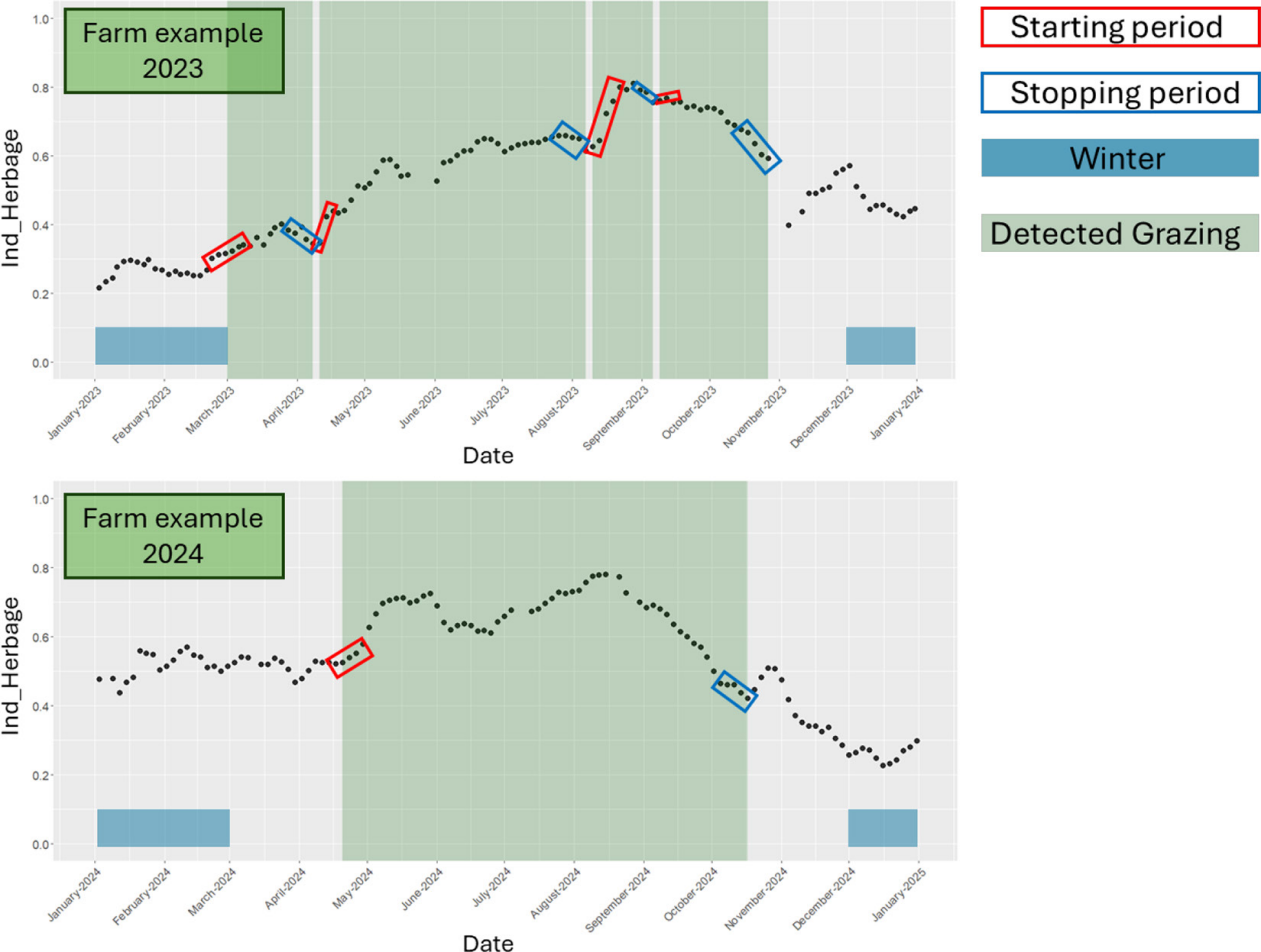
Evolution of the Ind_Herbage indicator and detection of the grazing period.

Based on observation of the detected pasture periods on the graphs of each farm, one minor correction is proposed in our approach. In the winter period, when herds in Wallonia cannot graze, increases in the signal were detected. These increases are most likely due to special feed supplementation such as flaxseed, which influences the content of some fatty acids in milk (Leduc et al., 2017). In the proposed algorithm, these need to be corrected. Therefore, the winter period is set to 0 by default. Even under the best weather conditions for some parts of the territory, we excluded the months of December, January, and February. This correction would need to be adapted to local conditions if the algorithm were tested on data from other regions. For DT1, a dataset for which we know with certainty that farm management included time on pasture, this correction did not change the final result in terms of detection above 120 d, but it did change the distribution of the number of days on pasture. The exact number of days is purely informative, as we could not validate the exact number of grazing days with the available data. Without the winter correction, in 2023 the number of days on pasture counted would be up to 343 d with a minimum of 120 and a mean of 256 d. The number of detected grazing periods would vary between 2 and 10 with a mean of 5.0. In 2024, the number of days on pasture counted would be up to 343 d with a minimum of 160 and a mean of 273 d, and the number of detected grazing periods would vary between 2 and 11 with a mean of 5.2.

Based on this approach, and with the winter correction, all 72 farms were detected as having more than 120 d on pasture, with 2 exceptions in 2023, leading to 97.2% correct attribution. As a small illustration of the impact of the algorithm's parameters, Table 1 presents results for 6 combinations of smoothing period and number of derivatives for the “start/stop” detection, for both 2023 and 2024. Considering potential heavy rains or weather changes, these numbers seem to be well aligned with the Belgian context, as they would separate 2 grazing periods detected by our algorithm. As expected, the number of derivative points required to start and stop grazing periods has a substantial impact on how often the signal is segmented. A sensitivity analysis like Table 1 would be required to assess the best set of parameters on a larger and more precise validation set. As presented in this study, it only allows for general assumptions regarding grazing screening sensitivity.

**Table 1:** Illustration of the variation of detected grazing days and grazing periods through several combinations of smoothing periods, and numbers of consecutive derivatives used to determine the beginning and end of a grazing period

Year	Parameter		Grazing days			Grazing periods		
	Smoothing period	Number of derivatives (start/stop)	Minimum	Maximum	Mean	Minimum	Maximum	Mean
2023	7	3	193	268	237.8	4	11	7.5
	7	4	110	268	212.3	2	8	4.5
	7	5	42	272	205.7	1	8	3.4
	14	3	164	266	240	3	9	6.1
	14	4	150	268	230.3	1	4.8	8
	14	5	150	268	225.8	1	6	3.7
2024	7	3	139	271	241.1	3	11	6.8
	7	4	126	271	221	1	9	4.6
	7	5	127	271	213	1	8	3.6
	14	3	136	269	247.7	3	8	5.3
	14	4	139	269	242.9	2	7	4.7
	14	5	165	269	235.4	2	7	4

The results with dataset DT2 present more contrast, probably due to the smaller size of the dataset and its precision. This allows us to draw attention to some specific cases. For example, the number of days per month detected for farm-month observations on pasture was 25.5 d (n = 32) versus 17.9 d (n = 34) for months tagged without grazing. In addition, due to the month-to-month precision, it sometimes occurs that the first and last months are categorized with a label but should contain both days on and off pasture.

In a more general view with DT3, in 2024, 96.6% of Walloon farms were detected as having more than 120 d on pasture. Results from the DairyClim survey (Dufrasne Isabelle and Françoise Lessire, 2017) show that 96% of farms graze for at least 4 mo per year, thus aligning with our results.

Regarding the potential impact of flaxseed on Ind_Herbage computation, DT2 did not allow us to draw significant conclusions, making it impossible to differentiate the farm effect from the additional flaxseed effect. Nevertheless, studies demonstrate that in certain cases, flaxseed ingestion modifies milk composition (Huang et al., 2022; Koçoğlu et al., 2025), and would therefore influence the computation of Ind_Herbage.

This study is based on several hypotheses. Most importantly, as explained in previous studies, a caveat is necessary: herbage consumption detected by the indicator can be induced by other feed compositions and does not necessarily mean that the cattle have been grazing on pasture. Another clear limitation of this study is the lack of “no-grazing only” farms, as such data were unavailable in the region. Nonetheless, this study lays the groundwork for effective use of potential indicators in practice to automate the task of verifying the number of grazing days and facilitate milk quality control.
